# Anti-Rheumatic Drugs for the Fight Against the Novel Coronavirus Infection (SARSCoV-2): What is the Evidence?

**DOI:** 10.31138/mjr.31.3.259

**Published:** 2020-09-21

**Authors:** Eleftherios Pelechas, Vassiliki Drossou, Paraskevi V. Voulgari, Alexandros A. Drosos

**Affiliations:** Rheumatology Clinic, Department of Internal Medicine, Medical School, University of Ioannina, Ioannina, Greece

**Keywords:** SARS-CoV-2, COVID-19, cytokine storm, ACE2 receptor, spike protein, IL-6, hydroxychloroquine, tocilizumab, colchicine

## Abstract

SARS-CoV-2 is a positive-sense single-stranded RNA virus that causes the COVID-19 infection. Spike proteins are the most important proteins found on its capsule using the host’s ACE2 receptors to invade respiratory cells. The natural course of the COVID-19 infection is variable, from asymptomatic to severe and potentially fatal. A small percentage of the severely infected patients will end up in an intensive care unit for ventilatory support. Elderly male patients with pre-existing medical conditions and smokers are at a disproportionate high risk to develop severe complications. Studies have shown that deaths occur due to a dysregulated immune system that overreacts, producing a plethora of cytokines, leading to the so-called “cytokine storm” phenomenon. In this direction, many drugs that are used in the everyday practice of Rheumatologists have been used. Indeed, pro-inflammatory cytokines such as the IL-1 and IL-6 have been shown to be the pivotal cytokines expressed, and anti-cytokine treatment has been tried so far with various results. In addition, hydroxychloroquine, an antimalarial drug, has been shown to reduce COVID-19 symptoms. Other drugs have also been used, such as intravenous pulses of immunoglobulins, and colchicine. Robust clinical trials are needed in order to find the suitable treatment. Current data indicate that hydroxychloroquine and cytokine targeting therapies may prove helpful in the fight of SARS-CoV-2 in appropriately selected patients.

## INTRODUCTION

SARS-CoV-2 (severe acute respiratory syndrome-related coronavirus) or colloquially known as “the novel coronavirus”, is a positive-sense single-stranded RNA (+ssRNA) virus responsible for the 2019–2020 COVID-19 pandemic outbreak that commenced in Wuhan, China.^1^ A COVID-19 infection usually causes a mild illness with symptoms mainly from the respiratory system, but it can develop to a serious and fatal disease with acute respiratory failure, shock, and severe damage to vital organs.^2^ Since its appearance, much has been heard and written about this disease. Most of the times, questions outweigh the reliable answers that can be given at this particular moment, especially regarding the treatment options that we already have in hand. There are several ongoing trials trying to give answers to the best possible therapeutic options in order to tackle the disease process, as currently there are no targeted therapeutics, and effective treatment options remain very limited.

## VIRAL INFECTIONS

In general, viruses are made up of a nucleus that includes their genetic material (DNA or RNA) and a peripheral capsule. The peripheral capsule constitutes various molecules or glycoproteins attached to its surface that are usually attached to target cells. Viruses do not have the necessary organelles and enzymes for protein synthesis, a vital process for their survival and reproduction. For this reason, in order to survive and reproduce, they are necessarily intracellular “parasites” depending on the biochemical equipment of the host cells. In addition, not every virus infects any cell, but rather shows a tropism for a target cell. For example, the hepatitis A virus infects the hepatocyte and the acquired immunodeficiency virus infects the CD4-T lymphocyte of the immune system.

The steps of taking over a host cell are the following five: 1. *attachment* of the virus to the target cell surface, 2. *penetration* and entry into the cytoplasm through the gateway used by the virus, 3. the virus opens its capsule (*uncoating*) and releases genetic material (DNA, RNA) into the cytoplasm, 4. genetic material is moved and incorporated into the nucleus of the host cell, where the synthesis of the viral mRNA and reproduction of the virus genome takes place (*replication*), 5. followed by the *assembly* of virus and genome proteins, and finally, the expression of the virus on the surface of the host cell which then is released.^3^

## SARS-CoV-2 INFECTION

The natural course of the coronavirus infection varies in the population. It has been observed that some factors that influence the course of the disease are the age, sex, and pre-existing conditions of the affected person. Thus, there are asymptomatic patients, patients with mild to moderate symptoms, and finally those with severe symptomatology that may also need medical support in an intensive care unit. The actual percentage of the positive to the virus people is not known. Those with mild to moderate symptoms represent approximately 80% of the positive tests and are advised to stay at home in order not to infect others.^4^ However, 10–15% will present severe symptoms, and 5–6% of them will need hospitalization in the intensive care unit, mainly for ventilatory support.^5^

### Transmission

According to the World Health Organization (WHO), respiratory infections can be transmitted through droplets of different sizes. When the droplet particles are >5–10μm in diameter, they are referred to as respiratory droplets (transmission occurs in close contacts through coughing or sneezing), while when they are ≤5μm in diameter, they are referred to as droplet nuclei (airborne transmission is possible as these particles have small diameter and can remain in the air for long periods of time). In the context of COVID-19, airborne transmission may be possible in specific circumstances (eg, administration of nebulized treatment).^6^

### Virulence by age, sex, and pre-existing medical conditions

It has been observed that an elderly male patient with pre-existing medical conditions is the typical patient that may develop severe complications.^7^ Why is the clinical response so variable amongst different populations? The answer lies within our immune system. The cells that are in the front line of our defence mechanisms are the polymorphonuclears (PMN) and macrophages (MΦ) which belong to the native immunity. Their main activity is phagocytosis. But, MΦ have cytotoxic activity and also act as antigen presenting cells (APCs). That means that they recognize the “invader”, and if they cannot destroy it, they present it to the T-lymphocytes, which in turn are activated effector T-cells and the B-lymphocytes and produce antibodies to kill and limit the spread of the virus within our body.

**Figure 1.**
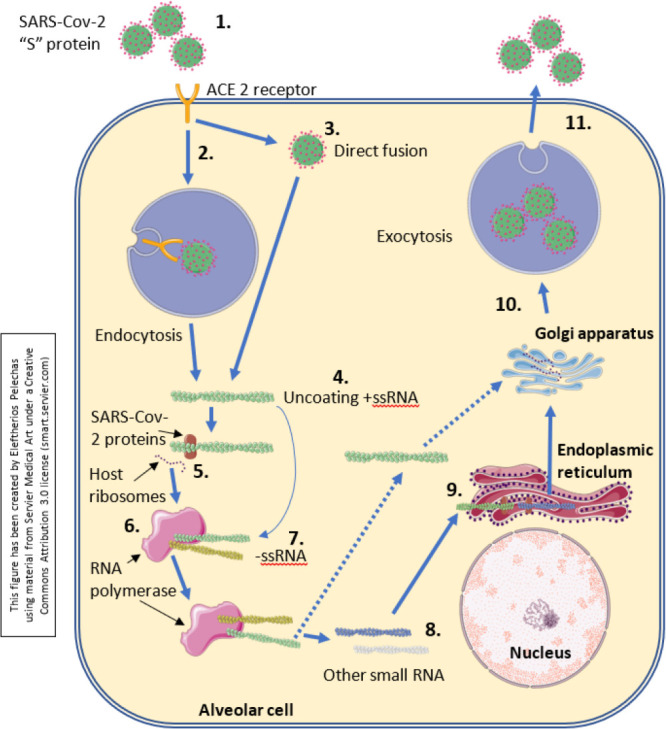
Invasion and replication of the SARS-CoV-2 within an alveolar cell. The virus targets and binds onto ACE 2 receptors on the surface of type 2 alveolar cells via its surface protein “spikes”.^1^ ACE 2 is needed for virus to gain entry inside the cell. This is a representation of the corona virus life cycle within the cell. Corona viruses will enter via endocytosis^2^ or direct fusion of the viral envelope with the host membrane.^3^ Once inside, the virus particle is uncoated, and its genome enters the cell cytoplasm.^4^ Since coronavirus has a single positive stranded RNA genome, it can directly produce its proteins and new genome in the cytoplasm by attaching to the host’s ribosomes.^5^ The host’s ribosomes will translate the viral RNA to make proteins that will make RNA polymerase.^6^ The RNA polymerase will read the positive strand of RNA of the virus again to make a negative RNA strand.^7^ The negative strand will be used by the RNA polymerase again to make a positive RNA strand as well other small positive RNA strands.^8^ These small RNA strands will be read by the host’s ribosomes again in the endoplasmic reticulum to help make the structural components of the virus.^9^ The endoplasmic reticulum will transfer these accessory and structural proteins into the Golgi apparatus where it will be packaged up with the positive RNA strand formed, to essentially form a new virus.^10^ These viruses are then released from the host cell by exocytosis through secretory vesicles.^11^ While the virus is self-replicating in the alveolar cells, it also damages it, and this will initiate the inflammatory response. Injured alveolar cells release interferons, cytokines as well as its intracellular components (not shown, continued in **Figure 2**).

**Figure 2.**
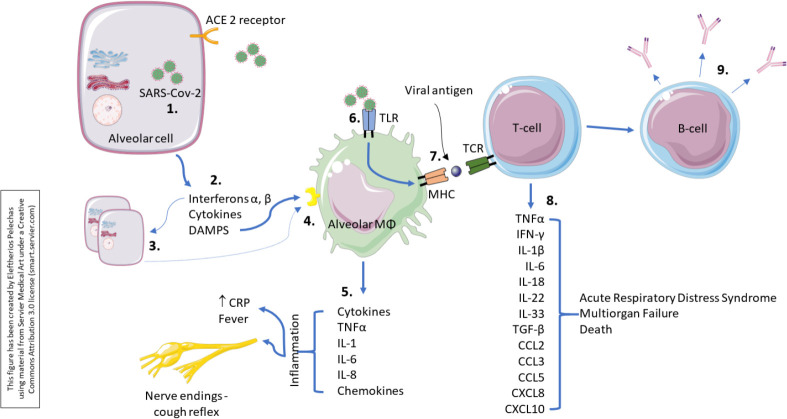
Immunological response to SARS-CoV-2. While the virus is self-replicating in the alveolar cells,^1^ it also damages it, and this will initiate the inflammatory response. Injured alveolar cells release interferons, cytokines as well as its intracellular components.^2^ Interferons α, β act in a paracrine manner, and have numerous effects on the surrounding cells preparing them for the ongoing infection. The primary function is to induce protection against viruses in neighbouring non-infected cells.^3^ Alveolar macrophages detect cell injury via damage-associated molecular patterns from the alveolar cells.^4^ They also respond to the cytokines released by injured alveolar cells. This causes the alveolar macrophages themselves to secrete cytokines such as TNFα, IL-1, IL-6, IL-8, as well as other chemokines.^5^ The inflammatory process occurring within the lung parenchyma stimulates nerve endings responsible for initiating the cough reflex. Thus, people often present with a dry cough early on. TNFα and IL-1β are pro-inflammatory cytokines and cause increased vascular permeability and increase in expression of adhesion molecules. This allows recruitment of more immune cells including neutrophils and monocytes. The alveolar macrophage can also detect the virus^6^ using its special receptors, called Toll-like receptors (TLRs). It can engulf the virus particles to phagocytosis, process it, and then present it on its surface.^7^ Studies have shown that the viral proteins can be presented. By presenting the viral proteins, specific T-cells may recognise them and mount an adaptive immune response^8^ consisting of T-cell activation and production of a plethora of proinflammatory cytokines and chemokines that may lead to a cytokine storm. In addition, B-cells or the plasma cells will then be activated and produce antibodies against the viral proteins.^9^

As we mentioned earlier, the new coronavirus is an RNA virus that has molecules (glycoproteins) on its capsule. The most important are the “Spike proteins”, having two subunits, the S1 and S2.^8^ These glycoproteins are used by the virus as a “key” assisting in the fusion of the viral and host cell membranes, and as a gateway they use the ACE-2 protein, an endogenous membrane protein than enables COVID-19 infection (**Figure 1**).^9^ This protein is the receptor for the angiotensin converting enzyme-2, which is found mainly in the cells of the respiratory system, MΦs, and other organs such as the heart, blood vessels, and kidneys. When the virus enters and unfolds its RNA in order to replicate, the MΦs stimulate the production of pro-inflammatory cytokines such as the interferon (IFN)α and β, tumour necrosis factor (TNF)α, interleukin (IL)-1 and IL-6. Viral infections mainly stimulate the production of IFNα and β, and thus they are called also “antiviral cytokines” because of their ability to prevent the virus from invading the body. In this way, MΦs can destroy the virus and prevent a further reproduction and assembly. Asymptomatic patients and those with mild symptoms belong to this category. The early innate response plays an important role in shaping the downstream adaptive immune response; however, an overactive innate immune response can also result in immune pathology and subsequent tissue damage.^10^ Arthralgias, myalgias, malaise, and fever are symptoms that appear due to the production of IFNα and β and other cytokines. However, in some patients the MΦ fails to kill the “invader” so it presents the virus to T-lymphocytes, which in turn activate and release IL-2 and IFN-γ and further activate MΦs but also B-lymphocytes for antibody production. At this stage, activation of MΦs downregulates the production of IFNα and β (which are antiviral cytokines), and upregulates the production of IL-6, IL-1 and TNF that leads to a further deterioration of the patient with high fever, persistent cough, shortness of breath, and possibly respiratory failure. In this case, the human organism reacts intensively and abruptly against the foreign “invader” with a “storm” by producing pro-inflammatory cytokines (cytokine storm), which can result in shock, acute respiratory failure and multiple organ failure (**Figure 2**). This category of patients with severe respiratory failure need increased care in intensive care units.^10^

Another question that arises is, why are the elderly and patients with chronic diseases usually affected? The simple answer could be that elderly patients also have an “aged” immune system that does not respond well enough to fight an infectious agent. In addition, most of the elderly patients have other comorbidities such as diabetes mellitus, chronic obstructive pulmonary disease, coronary artery disease, etc. Under these circumstances, the already deteriorated immune system has a “disease front” and tries to defend itself against these diseases by further reducing its overall activity and capability of fighting an infection.

Smokers make up another category of high-risk patients. It is well known that smoking causes chronic inflammation in the lungs, so the immune system has an “a priori disease front” by constantly defending itself against chronic inflammation. On the other hand, smoking causes overexpression of ACE-2 molecules in lung cells, which is the gateway for the SARS-CoV-2 virus, and in this way, even more virus molecules enter the cells. The conclusion is that the immune system of the elderly with co-existing chronic diseases that were or are smokers is not working adequately, and the defence mechanisms are impaired, so they are more easily affected and may experience a severe disease course.

## CURRENT AND EMERGING TREATMENTS

There is a phenomenal response from scientists around the globe to the ongoing coronavirus pandemic with a great scale, high-quality research effort towards a treatment for COVID-19. Interestingly, and almost intriguingly, most of the proposed treatment options by the infectious disease specialists include drugs that are being used in the everyday clinical practice by rheumatologists. In **Table 1**, a list with some of the ongoing trials is presented.

**Table 1.** Clinical trials in development for the treatment of SARS-CoV-2.

**Drug**	**Trial**	**Country (nr. of patients)**	**Completion date**	**Trial Number**
HCQ, AZN	Anti-coronavirus therapies to prevent progression of coronavirus disease (COVID-19) Trial (ACT COVID19)	International – Canada (1500)	30 September 2020	NCT04324463
HCQ	Post-exposure Prophylaxis / Preemptive Therapy for SARS-Coronavirus-2 (COVID-19 PEP)	Canada, US (3000)	21 April 2020	NCT04308668
HCQ	Efficacy and Safety of Hydroxychloroquine for Treatment of Pneumonia Caused by 2019-nCoV (HC-nCoV)	China (30)	25 February 2020 (Completed – no results posted)	NCT04261517
HCQ	Efficacy of hydroxychloroquine in patients with COVID-19	China (62)	28 February 2020 (completed with results in pre-print)	ChiCTR2000029559
HCQ	Hydroxychloroquine post exposure prophylaxis for coronavirus disease (COVID-19)	US (1600)	1 March 2021	NCT04318444
HCQ, AZN	Safety and efficacy of hydroxychloroquine associated with azithromycin in SARS-CoV2 virus (Coalition Covid-19 Brasil II)	Brasil (440)	30 August 2020	NCT04321278
TCZ (IL-6), favipiravir	Favipiravir combined with tocilizumab in the treatment of corona virus disease 2019	China (150)	1 May 2020	NCT04310228
Lopinavir-ritonavir, anakinra (IL-corticosteroids, 1), macrolides, interferon	Randomized, embedded, multifactorial adaptive platform trial for community-acquired pneumonia (REMAP-CAP)	Canada, Australia and countries (no 12 European maximum nr)	End April – Early May 2020	NCT02735707
Emapalumab, anakinra (IL-1)	Efficacy and safety of emapalumab and anakinra in reducing hyperinflammation and respiratory distress in patients with COVID-19 infection	Italy (54)	1 July 2020	NCT04324021
SAR (IL-6)	Cohort multiple randomized controlled trials open-label of immune modulatory drugs and other treatments in COVID-19 patients – Sarilumab Trial – CORIMUNO-19-SARI (CORIMUNO-SARI)	France (180)	26 March 2021	NCT04324073
SAR (IL-6)	Evaluation of the efficacy and safety of sarilumab in hospitalized patients with COVID-19	US (400)	16 March 2021	NCT04315298
TCZ (IL-6)	A study to evaluate the safety and efficacy of tocilizumab in patients with severe COVID-19 Pneumonia (COVACTA)	US (330)	31 August 2021	NCT04320615
TCZ, SAR	Anti-IL-6 treatment of serious COVID-19 disease with threatening respiratory failure (TOCIVID)	Denmark (200)	1 June 2021	NCT04322773
Colchicine	Colchicine coronavirus SARS-CoV2 Trial (COLCORONA) (COVID-19)	Canada (6000)	1 September 2020	NCT04322682
Colchicine	The Greek Study in the Effects of Colchicine in Covid-19 cOmplications Prevention (GRECCO-19)	Greece (180)	Not recruiting yet	NCT04326790
Immunoglobulin	The efficacy of intravenous immunoglobulin therapy for severe 2019-nCoV infected pneumonia	China (80)	30 April 2020	NCT04261426

HCQ: hydroxychloroquine; AZN: azithromycin; TCZ: tocilizumab; SAR: sarilumab.

### Hydroxychloroquine

**Figure 3.**
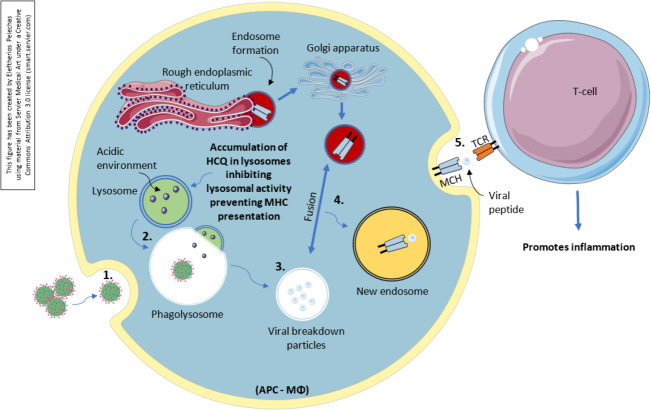
Antigen-processing onto macrophage/APC and the action of hydroxychloroquine. An antigen-presenting cell takes up the pathogen (SARS-CoV-2) end engulfs it with phagocytosis.^1^ Lysosomes that contain acids create an acidic environment and fuse with the phagocytosed “invader”, creating the phagolysosome which has an acidic environment as well.^2^ Because of the acidic environment, the content of the phagolysosome breaks down, leaving various particles that will be processed as antigens.^3^ In the meantime, within the endoplasmic reticulum the ribosomes are synthesizing MHC and allow the formation of an endosome which passes also through the Golgi apparatus to form a new endosome. The viral breakdown particles will then fuse with the new endosome and the particles will bind onto the groove of the MHC.^4^ Finally, the antigen presenting cell will express it on its self-surface.^5^ Hydroxychloroquine accumulates in lysosomes, raising the pH establishes a non-acidic environment, and inhibits the lysosomal activity. In this way, it prevents the MHC presentation to the surface of the antigen-presenting cell.

An example is hydroxychloroquine (HCQ). This drug has been used in rheumatology for more than 50 years. Its use is for the treatment of rheumatoid arthritis (RA) and systemic lupus erythematosus (SLE). It was first used against malaria (because it is a derivative of quinine) and then in rheumatic diseases. Due to the fact that many patients had side effects on chloroquine, HCQ was developed with fewer side effects, and is currently used in rheumatology. We do not know the exact mechanism of action to the virus of this drug. But we do know that HCQ inhibits the attachment of the antigens of a microorganism to MΦ cells due to inhibition of Toll-like receptors (TLRs). Another effect of the drug is that it helps MΦs to destroy the “invader” in their cytoplasm through the action of lysosomes. Finally, it prevents the presentation of the “invader” by MΦs in T-lymphocytes, suppressing the molecules of the Major Histocompatibility Complex (MHC) that present it (**Figure 3**). Could the above mechanisms of HCQ block the virus, prevent it from multiplying and spreading? *In vitro* studies showed that HCQ blocks viral replication by inhibition of cell entry of SARS-CoV-2 and prevents virus-cell fusion by interfering with glycosylation of ACE2 receptor and its binding with spike protein.^10^ This remains to be proven, and there are ongoing trials in this direction (**Table 1**). If the drug eventually proves useful, it should be given to patients very early to reduce the extent of the disease. However, we do not know the effective dose of the drug as well as the overall duration that should be administered.

Chen et al. conducted a randomized, parallel-group clinical trial in China with 62 patients. 31 patients were assigned to receive an additional 50day HCQ (400mg/d) treatment. They concluded that the use of HCQ could significantly shorten the time to clinical recovery (TTCR) and promote the resolution of pneumonia.^11^ In a recent open-label, non-randomized clinical trial, Gautret et al., despite the small sample size of the patients, showed that HCQ is significantly associated with viral load reduction/disappearance in COVID-19 patients and its effect is reinforced by azithromycin.^12^

The efficacy of different dosing regimens, alone or in combination with azithromycin, has been tested and compared to standard treatment or no therapy.^13–15^ Although HCQ in combination with azithromycin ended up in more frequent cases of QTc prolongation, arrhythmias and cardiac arrest in hospitalized patients.^13–16^

### Interleukin inhibitors

Two drugs that have similar action are being used, tocilizumab (TCZ)^17^ and sarilumab (SAR).18 These drugs are monoclonal antibodies that inhibit the IL-6 receptor. Rheumatologists have great experience using these drugs in the intravenous or the subcutaneous forms. Their therapeutic indications include the treatment of RA, systemic juvenile idiopathic arthritis (sJIA), juvenile idiopathic polyarthritis (pJIA), giant cell arteritis (GCA), but they are used also in cases of macrophage activation syndrome (MAS). In the above diseases, IL-6 is the pivotal cytokine in their pathogenesis, and in the same way, infection with the SARS-CoV-2 virus may lead to overexpression of this cytokine, leading further to a cytokine storm.^19–21^

On March 3, China approved the use of TCZ for coronavirus patients on the grounds that patients with serious lung damage showed increased levels of IL-6. However, there was no clinical trial evidence yet that the drug would be effective on coronavirus patients. On the other hand, there are some recently published case reports pointing out that TCZ may be a potential therapeutic option for the COVID-19 patients. Michot et al. reported successful treatment of a patient with respiratory failure related to COVID-19. The patient was immunosuppressed because of his cancer. The authors state that patients with hyperinflammatory pulmonary symptoms that are associated with a cytokine storm, may find IL-6 inhibitor TCZ a promising therapy.^22^ Zhang et al. used TCZ in a multiple myeloma patient with success. The patient developed multiple ground-glass opacities on the grounds of COVID-19 infection, and on day 19, reported that the range of ground-glass opacities had obviously decreased after use of TCZ.^23^ Mihai et al*.* did not report a successful treatment of COVID-19 infection, but they claimed that TCZ may have helped their patient with the duration of the symptoms and the evolution of the COVID-19 as she was a high-risk patient (systemic sclerosis with interstitial lung disease and pre-existing lung involvement <20% and diabetes mellitus type 2). Furthermore, the authors reported that IL-6 blocking treatment given for chronic autoimmune diseases may even prevent the development of severe COVID-19.^24^ Luo et al*.* retrospectively assessed 15 patients with COVID-19 (2 moderately ill, 6 seriously ill and 7 critically ill). They concluded that not only does TCZ appear to be an effective treatment option in COVID-19 patients with risk of cytokine storm, but also to repeat the dose of TCZ on critically ill patients with elevated IL-6.^25^ Finally, the latest bibliographic evidence from several authors such as Sciascia et al.^26^ Campins et al.,^27^ and Conrozier et al.,28 points out that TCZ is a safe choice even at high dosages and with repeated administration protocols in combination or without the use of pulse steroid therapy.^27^

Other monoclonal antibodies such as the IL-1 (anakinra)^29^ and TNFα (infliximab, adalimumab etc) may be useful in the control of the severe inflammatory responses. The IL-1 inhibitor anakinra is used for the treatment of RA (albeit not currently), the cryopyrin-associated periodic syndromes (CAPS), and Still’s disease, but it can also be administered in patients with gout flares (not controlled by conventional drugs), and relapsing polychondritis.

All the above are drugs that target the pro-inflammatory cytokines produced when the MΦ cell is over-activated. As we already mentioned, it is these cytokines that cause the most serious damage resulting in septic shock and multiple organ failure. Monteagudo et al. retrospectively analysed a chart of five patients with MAS, another form of cytokine storm. They postulate that continuous infusion of intravenous anakinra may result in rapid serologic and subsequent clinical improvement in adult patients with MAS and this method should be considered also for treating the cytokine storm in patients with COVID-19.^30^ Moutsopoulos HM observed that a 70-year-old woman who was diagnosed with cryopyrin-associated periodic syndrome has been treated with canakinumab 10 days prior of tested positive for SARS-CoV-2. Given the patient’s age and immunomodulation due to the IL-1 blockade, she would be classified as high risk for developing a serious COVID-19 infection. However, the patient’s clinical picture was very mild highlighting that cytokine blockade may protect patients.^31^

### Janus Kinase inhibitors

Baricitinib has been proposed as a potential treatment for SARS-CoV-2 infection considering its inhibitory activity both on cytokines production and on Coronavirus endocytosis.^32,33^ Indeed, it has been shown that patients employed on this drug had a significant improvement of clinical and laboratory parameters. Cantini et al. in an open-label clinical trial including 24 patients with moderate COVID-19 infection reported that none of the patients required intensive care unit support, and most were discharged within 2 weeks of enrolment. Also, no adverse events were reported.^34^ On the other hand, Dr. Stebbing observed that the infective profile of Janus Kinase inhibitors (JAK1/2) might be not safe enough.^32^ These results should be also taken into account for future trials regarding which kind of JAK inhibitors will be the best in COVID-19.

### Other drugs

In addition to the above, intravenous pulses of immunoglobulins have been also used. The action of immunoglobulin is due to the fact that it binds to the virus and transmits the virus to PMN cells and MΦ, where the corresponding receptors of IgG immunoglobulin (FcγR) are present, thus, destroying the virus through phagocytosis. Cao et al*.* reported 3 patients with severe COVID-19 who received high-dose intravenous immunoglobulin with satisfactory recovery,^35^ and based on this observation, they pointed out that randomised studies of high-dose immunoglobulins should be considered.

Colchicine, a drug used mainly for gout, inhibits the migration of PMN cells to the site of inflammation. Furthermore, another mechanism is that of non-selective inhibition of NLP3 inflammasome, an innate immune signalling complex which is the key mediator of IL-1 family cytokine production, by downregulating the severe inflammatory response and this should aid the critically ill COVID-19 patients. Currently, the GRECCO-19 (NCT04326790), a prospective, cluster randomized, open-labelled, controlled study, and the COLCORONA study may shed light of the usefulness of Colchicine.^36^

Corticosteroids, although frequently used in clinical practice, especially in critical cases where they may control the inflammatory response related to the cytokine storm, are highly controversial due to the results of two meta-analyses.^37,38^ These meta-analyses concluded that corticosteroids might be associated with higher mortality, longer length of stay, a higher rate of bacterial infection, hypokalaemia,^37^ and a delay in the virus clearing without clearly improving survival or preventing intensive care unit admissions.^38^ Finally, Ledford H in a large trial (RECOVERY trial) supports that dexamethasone cuts deaths by one-third among patients critically ill with COVID-19.^39^

## IMPACT OF THE SARS-CoV-2 INFECTION IN PATIENTS WITH SYSTEMIC AUTOIMMUNE AND CHRONIC INFLAMMATORY DISEASES

As seen above, many anti-rheumatic drugs are being used or have been tried in patients with COVID-19. The pivotal question that arises is what is the impact of the SARS-CoV-2 infection in patients with systemic autoimmune and chronic inflammatory diseases that are already undergoing treatment with one or more of these drugs? Are they protected against SARS-CoV-2, or does their immunocompromised state put their life in danger?

The truth is, that data is limited. Despite that, several Rheumatology Societies have already released statements to promote health care of rheumatic patients during the current pandemic including the American College of Rheumatology (ACR), the European League against Rheumatism (EULAR), but also several national Societies such as the Italian and the Greek Rheumatology Societies.^40–43^ Up to date, in most cases, although more than half of infected patients experienced SARS-CoV-2-related pneumonia, the clinical course of the infection was relatively favourable such as the case we already mentioned by Moutsopoulos HM in a patient treated with canakinumab.^31^ Patients with autoimmune diseases may have different responses to the SARS-CoV-2 infection due to the concomitant treatment with immunosuppressive drugs. The drugs used by those patients may reduce, at least partly, the aggressiveness of the SARS-CoV-2 or may modulate the excessive host immune activity.^44^ On the other hand, due to the limited data for this category of patients, the management and follow-up should be very close, but we must keep in mind that they should avoid direct access to the hospital using the facilities of telemedicine wherever feasible.

## CONCLUSION

The ongoing COVID-19 pandemic revealed the global unpreparedness of the healthcare systems to respond promptly and efficiently to a new clinical entity. Even countries with the most advanced healthcare systems seem to be in dire straits, already counting thousands of deaths. The international scientific community is now trying to find the best therapeutic options for those suffering from severe complications of the COVID-19. There is a plethora of drugs that are being used with different results so far, but the lack of randomised controlled trials makes it difficult to choose the best treatment scheme. In this article, we have not included trials with antiviral agents, trying to focus on the already available anti-rheumatic drugs that exist to date. So far, the only efficient way of stopping the spread of this potentially fatal virus is to avoid being exposed to it. To achieve that, simple measures such as personal hygiene and avoiding close contact with people who are sick are the most important ones.

All the above, indicate that HCQ and cytokine targeting therapies may prove helpful in the fight of SARS-CoV-2 in appropriately selected patients. Rheumatologists have been using the above drugs for a long time and could prove useful in the battle of coronavirus, especially to those patients that develop severe complications and the so-called “cytokine storm”.

Finally, we as rheumatologists must be in the forefront dealing with the SARS-CoV-2 infection as we are already aware of other viral infections that produce systemic manifestations “manipulating” our immune system, such as cryoglobulinemia and hepatitis C virus^45^ or polyarteritis nodosa and hepatitis B virus infection.^46^ The paradigm of those two clinical entities (cryoglobulinemia and polyarteritis nodosa) relies on the fact that we do not only use antiviral agents, but also immunosuppressive therapies in order to control severe manifestations.^47^ In fact, immunosuppressive therapies may be used prior to starting the antiviral agents in order to appease the severe systemic manifestations.
